# A meta-analysis of BRF2 as a prognostic biomarker in invasive breast carcinoma

**DOI:** 10.1186/s12885-020-07569-8

**Published:** 2020-11-11

**Authors:** Stephanie Cabarcas-Petroski, Patricio I. Meneses, Laura Schramm

**Affiliations:** 1 Biology Department, Pennsylvania State University, Beaver Campus, Monaca, PA USA; 2 Department of Biological Sciences, Fordham University, Bronx, NY USA; 3 Department of Biological Sciences, St. John’s University, Queens, NY USA

**Keywords:** RNA polymerase III, TFIIIB, BRF2, Cancer, Prognostic marker

## Abstract

**Background:**

Deregulation of the RNA polymerase III specific TFIIIB subunit BRF2 occurs in subtypes of human cancers. However, correlations between BRF2 alterations and clinical outcomes in breast cancer are limited. We conducted this review to analyze BRF2 alterations in genomic data sets housed in Oncomine and cBioPortal to identify potential correlations between BRF2 alterations and clinical outcomes.

**Methods:**

The authors queried both Oncomine and cBioPortal for alterations in BRF2 in human cancers and performed meta-analyses identifying significant correlations between BRF2 and clinical outcomes in invasive breast cancer (IBC).

**Results:**

A meta cancer outlier profile analysis (COPA) of 715 data sets (86,733 samples) in Oncomine identified BRF2 as overexpressed in 60% of breast cancer data sets. COPA scores in IBC data sets (3594 patients) are comparable for HER2 (24.211, median gene rank 60) and BRF2 (29.656, median gene rank 36.5). Overall survival in IBC patients with BRF2 alterations (21%) is significantly decreased (*p* = 9.332e-3). IBC patients with BRF2 alterations aged 46 to 50 have a significantly poor survival outcome (*p* = 7.093e-3). Strikingly, in metastatic breast cancer, BRF2 is altered in 33% of women aged 45–50. BRF2 deletions are predominant in this age group.

**Conclusion:**

This study suggests BRF2 may be an prognostic biomarker in invasive breast carcinoma.

**Supplementary Information:**

The online version contains supplementary material available at 10.1186/s12885-020-07569-8.

## Background

In 2020, the leading sites for new cancers and deaths in the United States (U.S.) will be breast, prostate, lung, and colorectal cancers [1]. For U.S. women, breast cancer is the second leading cause of cancer-related deaths, second to lung [2]. Cancer mortality has continued to decline from its peak in 1991; there has been a consistent rise in breast cancer rates since 2004, approximately 0.3% per year [1]. An estimated 276,480 new cases of invasive breast cancer (IBC) are expected to be diagnosed in 2020 [2].

A common characteristic of many cancers is deregulated cell proliferation. In eukaryotic cells, regulation of cell proliferation is controlled, in part, by three distinct RNA polymerases (pol) [3]. RNA pol I regulates rRNA synthesis; RNA pol II transcribes mRNA and some small untranslated RNA molecules involved in RNA processing [3]. Small untranslated RNA molecules involved in processing and translation, regulating the biosynthetic capacity of a cell, are transcribed by RNA pol III. Accurate initiation of transcription by eukaryotic RNA polymerases require general and gene-specific transcription factors [3]; recruitment of promoter-specific transcription factors regulates cell proliferation [4].

RNA pol III recognizes both gene- internal and external promoters and requires two forms of TFIIIB [3, 5]. RNA Transcription from gene internal (type 2) RNA pol III promoters requires a form of TFIIIB, which includes TBP, BDP1, and BRF1. In the case of transcription from type 3 RNA pol III promoters, the form of TFIIIB required includes TBP, BDP1, and BRF2 [5, 6]. The TFIIB family members BRF1 and BRF2 (TFIIB related factors) have related N-terminal zinc ribbons and core domains [3]. BRF1 and BRF2 have unrelated c-terminal domains conferring RNA pol III promoter recognition specificity [7].

Atypical RNA pol III transcription is a common feature of many cancer types. TFIIIB activity is targeted both directly and indirectly by a variety of oncogenes and tumor suppressors [8, 9]. For example, the oncogenes MAP kinase ERK and MYC [10, 11] stimulate TFIIIB activity in vitro. The tumor suppressors p53 [11, 12], PTEN [13, 14], BRCA1 [15], the Retinoblastoma protein (RB) [11], and the Rb family members p107 and p130 [16] inhibit TFIIIB activity.

The TFIIIB subunit BRF2 is differentially expressed in cancer cells [17] and overexpressed in a subset of cancer patients [8]. BRF2 is regulated by chemopreventive agents in cancer cells and a mouse model [18, 19]. Amplification of BRF2, 8p11.23, and chromosome 8 frequently occurs in somatic breast cancer [20]. Previously, BRF2 expression in human cancers using the Oncomine 3.0 database [21] analyzed transcriptome data across 18,000 gene expression microarrays and 35 cancer subtypes and identified BRF2 overexpression in patients with gastric, kidney, melanoma, and lung cancers [8]. Using bioinformatic approaches [21], we demonstrated BRF2 overexpressed in 154 IBC (*p* = 3.53E-10) [19]. Specifically, BRF2 overexpression in invasive ductal carcinomas (IDC) was the most significant (*p* = 2.17E-21) [19]. Recently, studies identified BRF2 as a prognostic marker of unfavorable survival for both squamous cell carcinoma (SqCC) (*p* = 0.007) [22] and non-small cell lung cancers (NSCLC) (*p* = 0.001) [23], as well as esophageal cancers (*p* = 0.009) [24]. Interestingly, BRF2 expression is an independent prognostic factor for overall survival (*p* = 0.014) and biomarker for progression-free survival (*p* = 0.014) for patients with esophageal squamous cell carcinoma (ESCC) undergoing three-field lymph-node dissection (3FLND) treatment [25]. Together, these data suggest a comprehensive analysis of human cancer data sets to identify BRF2 alterations and clinical outcomes is warranted.

In this study, we performed a meta-analysis of patient data from both Oncomine and cBioPortal databases [21, 26] to analyze BRF2 alterations in human cancers, with a focus on breast cancer. Furthermore, we evaluated BRF2 as a potential prognostic biomarker in breast cancer. Herein, we report for the first time, a possible role for BRF2 as a prognostic marker in invasive breast cancer (IBC). Specifically, the data presented herein suggest BRF2 could serve as a prognostic marker for IBC in patients aged 46 to 50.

## Methods

### Oncomine analyses and data sets

We performed a comprehensive query of the Oncomine Research Edition, a cancer *microarray database* and web-based *data*-mining platform [21] containing 715 data sets (86, 733 samples), to determine the frequency of BRF2 mutations, alterations in BRF2 DNA copy number, and BRF2 gene expression in human cancers. The data sets analyzed are available at http://www.oncomine.org. A disease summary analysis for BRF2 was performed under stringent conditions as requiring a threshold *p*-value of 1E-4, a fold-change of 2 for BRF2 gene expression compared to the controls, and a gene rank percentile of top 10%. For BRF2 overexpression in specific data sets, sample numbers and *p*-values are indicated in the figure legends. The Outlier analysis normalizes expression data making the median of each expression feature across all samples 0.0 and the mean absolute deviation 1.0 [21]. Genes are then ranked based on their value at the 75th, 90th, or 95th percentiles in the Outlier analysis. In this study, Outlier analysis was performed using stringent criterion using a threshold *p*-value of 1E-8, a threshold fold change in gene expression to 20, and a threshold gene rank to the top 1% of genes. The Curtis Breast 2 [27] and TCGA Breast 2 [28] data sets, both with over 1000 patients’ samples, were restricted to the 95th percentile and queried to determine the median gene rank and COPA score for BRF2 and HER2. The Oncomine™ Platform (Thermo Fisher, Ann Arbor, MI) was used for analysis and visualization. BRF2 expression and copy number frequency in human cancers were analyzed and visualized using Oncomine Power Tools (http://www.powertools.oncomine.com).

### cBioPortal analyses and data sets

To complement the BRF2 expression analysis performed using Oncomine, we used the cBioPortal [26] to look at BRF2 alterations and clinical outcomes in the IBC (TCGA, Firehose Legacy) data set [28]. Cancer computational biologists and bioinformatics experts maintain the cBioPortal. Recently the cBioPortal has partnered with the Hyve bridging the gap between academic and industry experts in bioinformatics platforms. The cBioPortal’s TCGA, Firehose Legacy Breast Invasive Carcinoma data set notes raw data was generated by the TCGA Research Network (https://www.cancer.gov/tcga) and source mutation data is pulled from the Broad Institute’s GDAC Firehose (https://gdac.broadinstitute.org/). In our current analysis of BRF2 alterations in IBC, we included the data available in cBioPortal’s TCGA, Firehose Legacy data set for analysis consisting of 960 patients with available mutation, CNA, and mRNA microarray expression (z-score threshold + 2.0). BRF2 alterations in the Breast Cancer METABRIC (Nature 2012 & Nature 2016) [29] data set analyzed included patients with mutation, CNA, and mRNA microarray expression (z-score threshold + 2.0). Overall Survival Kaplan-Meier Estimate, and disease-free survival, are calculated within the cBioPortal and logrank *p*-value is provided, where available. Queries of the cBioPortal were performed in September 2019, October 2019, November 2019, January 2020, February 2020, and March 2020.

## Results

**Fig. 1:**
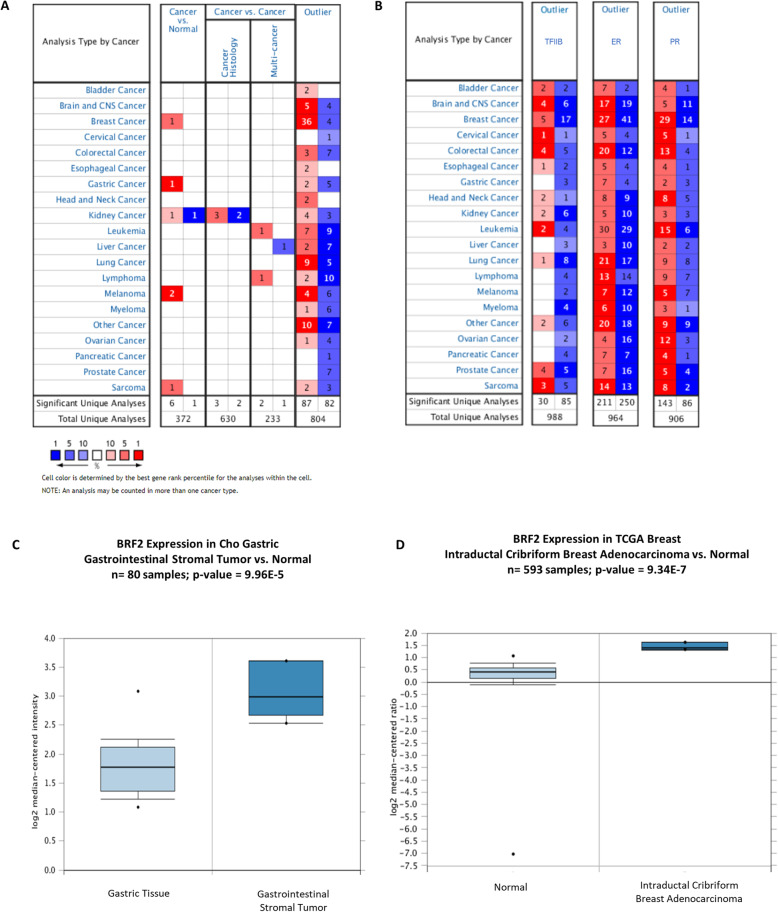
Oncomine disease summary for BRF2. Oncomine 4.5 was queried for (**a**) BRF2 expression in 715 data sets (83,733 samples) based on: cancer type, cancer versus normal, cancer versus cancer including histology and multi-cancer analysis types, and outlier analyses. Red shading of boxes denotes gene overexpression and blue shading represents decreased gene expression. The levels of expression are based on the gene rank percentile of 10%. This disease summary was performed using a criterion of a 2-fold change for gene expression and a *p*-value of 1E-4. BRF2 (**a**) is overexpressed in the following cancer vs normal data sets: breast, gastric, kidney, melanoma, and sarcoma cancers. In the cancer vs cancer data sets, BRF2 is overexpressed in kidney cancer (cancer histology data set), leukemia and lymphomas (multi-cancer) data sets. The outlier analysis demonstrates that BRF2 is both over- and under-expressed across the analyzed cancers. **b** Using the same stringent criterion, outlier analyses for estrogen receptor alpha (ER) (ESR1) and the progesterone receptor (PR), both categorized as biomarkers in breast cancer [30], were performed In addition, an Oncomine outlier analysis of TFIIB demonstrates over expression in 5 of 67 (0.07%) and underexpressed in 17 of 67 (25%) significant unique analyses of breast cancer data sets. The data sets analyzed during the current study are available at www.oncomine.org. Queries of Oncomine were performed in September 2019, October 2019, November 2019, January 2020, February 2020, and March 2020. (**c**) BRF2 is significantly overexpressed in Cho Gastric Stroma Data set [31], *n* = 80 samples and *p* = 9.965E-5. There is a 2.4-fold increase in BRF2 mRNA expression gastrointestinal stromal tumors as compared to controls. **d** BRF2 is significantly overexpressed in the TCGA Breast Cancer Data set [28], *n* = 593 samples and *p* = 9.34E-7. There is a 2.3-fold increase in BRF2 mRNA expression in Intraductal Cribriform Breast Adenocarcinoma, a form of invasive breast cancer, as compared to controls. The data sets analyzed during the current study are available at www.oncomine.org. Queries of Oncomine were performed in September 2019, October 2019, November 2019, January 2020, February 2020, and March 2020

We queried Oncomine, containing 715 data sets (86,733 samples), to determine the frequency of BRF2 alterations in human cancers [21]. The disease summary analysis for BRF2 (Fig. 1a) identified 7 significant unique analyses of BRF2 expression out of 372 unique analyses in studies comparing cancer versus normal tissue. In breast, gastric, kidney, melanoma and sarcoma cancer data sets, BRF2 is highly overexpressed, compared to normal tissue as represented by the red cells in Fig. 1a. In a subset of kidney cancer, 1 significant unique analysis shows that BRF2 is underexpressed in comparison to normal tissues, as represented by the blue cells in Fig. 1a. This BRF2 disease analysis was performed using a threshold *p*-value of 1E-4, a fold-change of 2 for BRF2 gene expression compared to the controls, and a gene rank percentile of top 10%.

In the cancer versus cancer data sets (Fig. 1a), BRF2 is both over- and under-expressed. Specifically, in the cancer histology data set for kidney cancer, BRF2 is both overexpressed (3 significant of 630 unique analyses) and underexpressed (2 significant of 630 unique analyses). Similarly, BRF2 is both over- and under- expressed in the multi-cancer data sets. BRF2 is overexpressed in both the leukemia (1 significant of 233 unique analyses) and lymphoma (1 significant of 233 unique analyses) data sets and underexpressed in a liver cancer (1 significant of 233 unique analyses) data set (Fig. 1a).

Within the disease summary analysis for a gene, the Oncomine Outlier tab reports the number of data sets in which the query gene had the highest-ranking Cancer Outlier Profile Analysis (COPA) score [21]. COPA is an effective method to analyze cancer expression data to potentially identify potential oncogenes that are differentially expressed in a subset of cancer data sets [21]. Using a threshold *p*-value of 1E-4, a 2 a fold-change for BRF2 gene expression compared to the controls, and a gene rank percentile of 10%, we demonstrate that BRF2 is both over- and under-expressed across the analyzed human cancers (Fig. 1a). BRF2 is specifically overexpressed in 36 of 60 (60%) and underexpressed in 4 of 60 (0.07%) of breast cancer data sets analyzed (Fig. 1a). To the best of our knowledge, this is the first report using a meta-analysis of 715 data sets (86, 733 samples) identifying BRF2 as overexpressed in 60% of breast cancer data sets in an Outlier analysis. To elucidate the potential significance of this observation, we performed an Outlier analyses for the founding member of the TFIIB family of genes, TFIIB [5], and two well characterized biomarkers in breast cancer, estrogen receptor alpha (ER) and the progesterone receptor (PR) [30] (Fig. 1b). Using the same stringent threshold criteria used in the outlier analysis for BRF2 (Fig. 1a) an outlier analysis of TFIIB demonstrated TFIIB overexpression in 5 of 67 (0.07%) and TFIIB underexpression in 17 of 67 (25%) breast cancer data sets analyzed (Fig. 1b). Outlier analyses for ER revealed overexpression in 27 of 67 (41%) and underexpression in 41 of 66 (62%) breast cancer data sets included.

In this same analysis, the PR [30] is overexpressed in 29 of 62 (47%) and underexpressed in 14 of 62 (23%) breast cancer data sets. Together, these data suggest the observed BRF2 overexpression in breast cancer warrant a more detailed investigation to determine if BRF2 alterations correlate with clinical outcomes.

We further queried the analyses presented in Fig. 1a to determine the extent of BRF2 overexpression in specific cancers. BRF2 overexpression has been detailed in melanoma, gastric, and kidney cancers, Fig. 1a and [8]. Fold change in BRF2 expression (cancer versus control) and sample numbers in melanoma, gastric and kidney cancers data sets remains unchanged as previously reported [8] and data is not shown. In addition, BRF2 is also significantly overexpressed in sarcoma (Fig. 1c), 1 significant of 5 (20%) unique analyses. BRF2 overexpression in sarcoma was observed in the Cho Gastric Stroma data set [31], *n* = 80 samples and *p* = 9.965E-5 (Fig. 1c). BRF2 mRNA expression was increased 2.4-fold in gastrointestinal stromal tumors as compared to controls (Fig. 1c). In addition, BRF2 is significantly overexpressed in the TCGA Breast Cancer data set [28], *n* = 593 samples and *p* = 9.34E-7 (Fig. 1d). There is a 2.3-fold increase in BRF2 mRNA expression in intraductal cribriform breast adenocarcinoma as compared to controls (Fig. 1d).

**Fig. 2:**
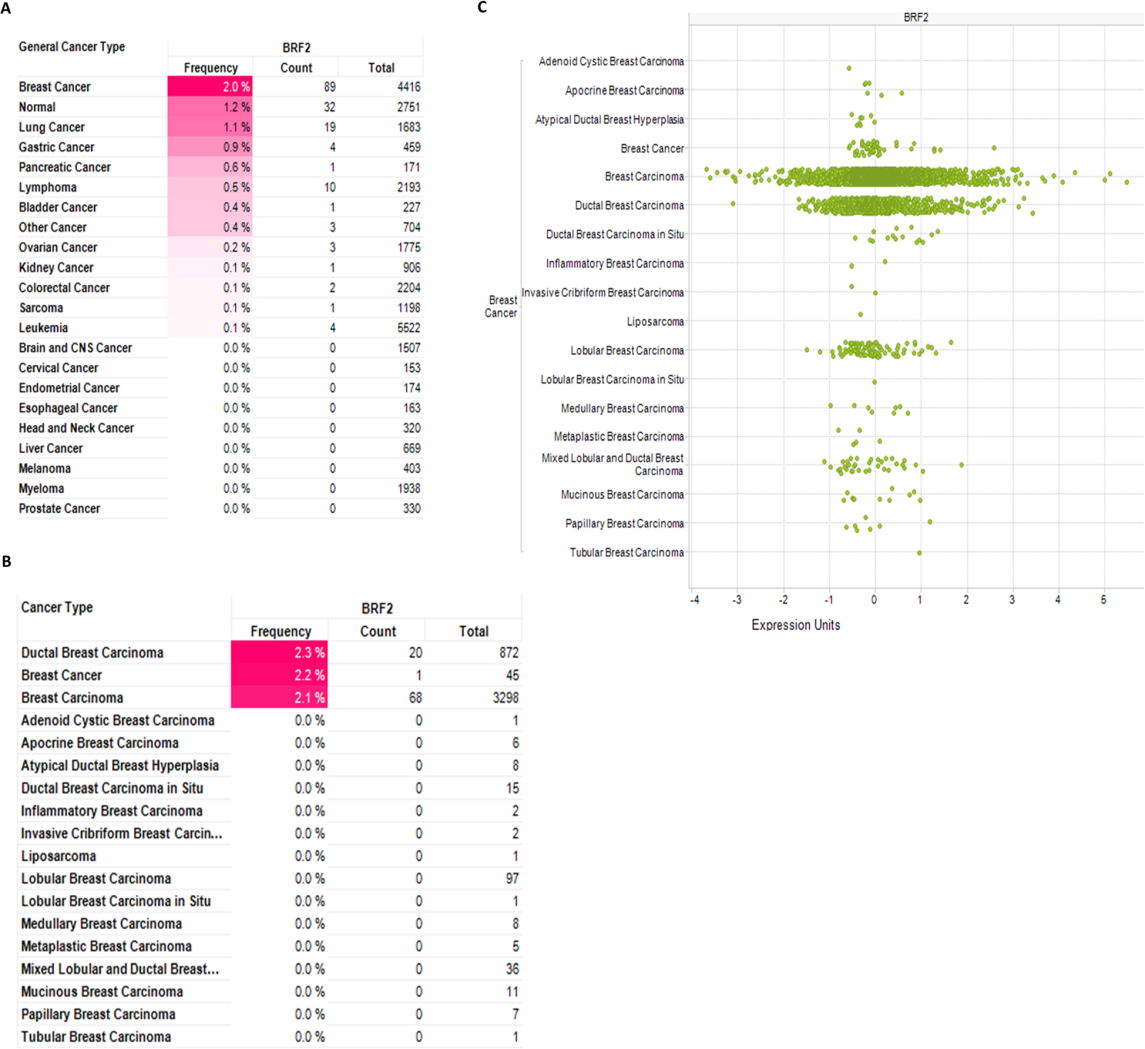
BRF2 is frequently overexpressed in ductal breast carcinoma. **a** BRF2 expression frequency by general cancer type. **b** BRF2 expression frequency by breast cancer sub-type. Red shading denotes BRF2 overexpression. **c** Scatterplot representing individual patient samples BRF2 expression across the expression value range on x-axis. The Oncomine Power Tools expression data from Affymetrix U133A, U133A 2.0, and U133 Plus 2.0 platforms is quantile normalized whereby the median is calculated across all samples and all genes. The data sets analyzed during the current study are available at https://powertools.oncomine.com/

The observed statistically significant overexpression of BRF2 in intraductal cribriform breast adenocarcinoma, p = 9.34E-7 (Fig. 1d), prompted a more detailed look at BRF2 alterations in subtypes of breast cancer (Fig. 2). Using the Oncomine Power Tools, we determined the frequency of BRF2 of overexpression to be highest in breast cancers (2.0%) as compared to lung (1.1%) cancer (Fig. 2a) where BRF2 has been identified as a novel lineage-specific oncogene in lung squamous cell carcinoma [32]. The highest incidence of BRF2 overexpression was observed in IDC, Fig. 2b. IDC accounts for approximately 80% of all breast cancer diagnosed [1]. Figure 2c is a scatterplot representing individual patient samples BRF2 expression across the expression value range on x-axis and confirms BRF2 is most frequently overexpressed in IDC in the data sets available in Oncomine. It is important to note, the Oncomine Power Tools expression data from Affymetrix U133A, U133A 2.0, and U133 Plus 2.0 platforms is quantile normalized whereby the median is calculated across all samples and all genes. The individualized IDC patient data presented in Fig. 2c permitted calculation of the average fold change for BRF2 expression as 1.22. In addition, 9% (135 of 1518 IDC patients) had an estimated fold change in BRF2 expression greater than 2.0. The estimated fold change in BRF2 expression ranged from 0.1 to 16.8 in 1518 patients diagnosed with IDC.

**Fig. 3:**
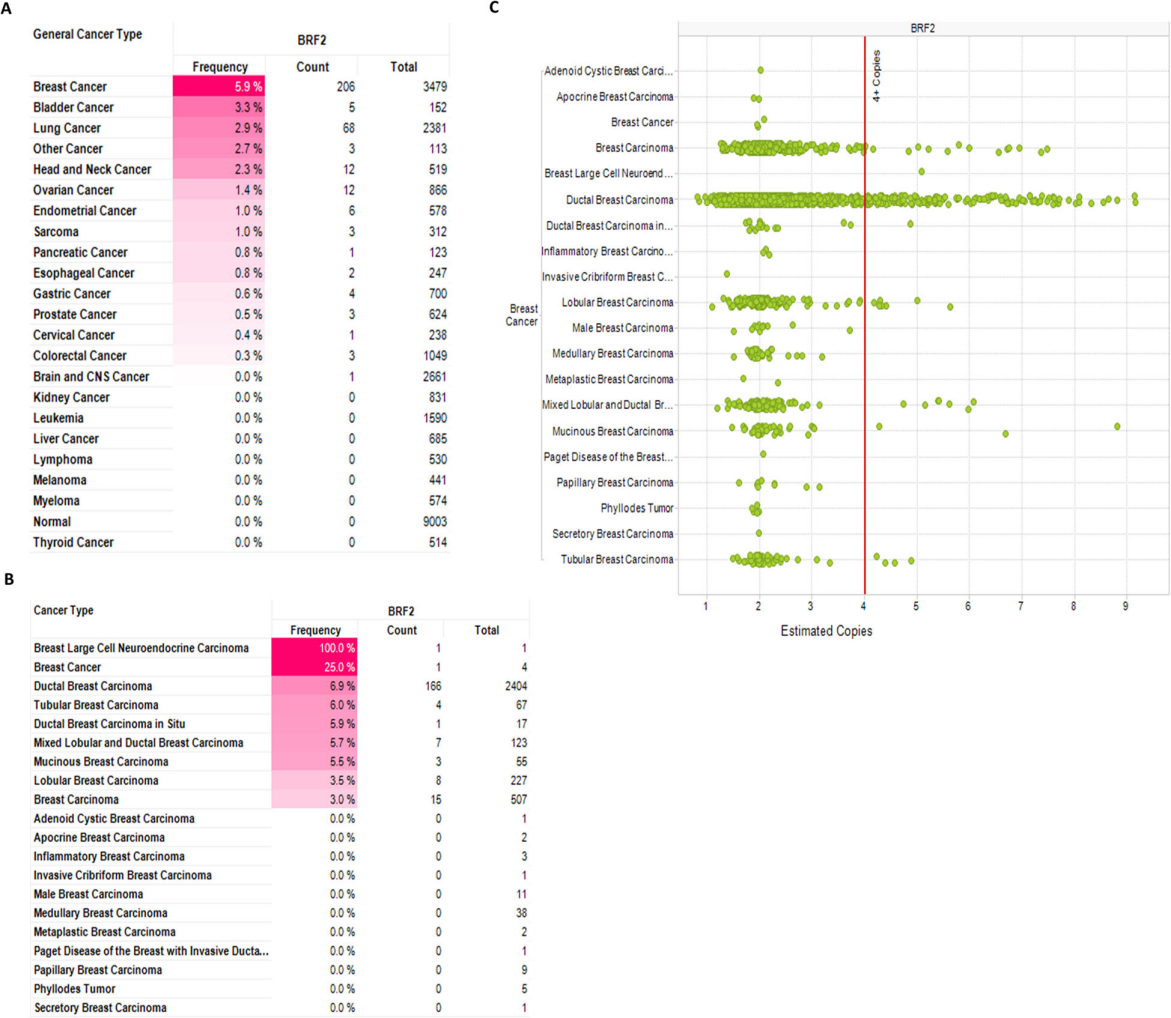
BRF2 DNA copy number is increased in ductal breast carcinoma. **a** BRF2 DNA copy frequency by general cancer type. **b** BRF2 DNA copy frequency by breast cancer sub-type. Red shading denotes increased BRF2 copy number. **c** Scatterplot representing individual patient BRF2 copy number value on x-axis. The data sets analyzed during the current study are available at https://powertools.oncomine.com/. Queries of Oncomine Power Tools were performed in September 2019, October 2019, November 2019, January 2020, February 2020, and March 2020

Chromosome 8 is frequently amplified in human breast cancer cell lines [33] and BRF2, located on chromosome 8p11.23, has been demonstrated to be amplified in breast cancer and identified as a potential oncogenic driver in breast cancer [33]. As such, we investigated the frequency of DNA copy events across human cancers (Fig. 3a). BRF2 copy number events occur most frequently in breast cancer, 5.9%, as compared to other human cancers analyzed (Fig. 3a). BRF2 copy number frequency is increased in 166 of 2404 (6.9%) of IDC patients (Fig. 3b). Figure 3c is a scatterplot of BRF2 estimated copy number events by breast cancer subtype. In Oncomine Power Tools, estimated DNA copies is computed as 2 * 2 ^, median-centered log2 copy number, assuming the median copy number is 2 copies per loci. From scatterplot of patient data presented in Fig. 3c, we calculated average estimated copy number for BRF2 in IDC as 2.28, and 42% (1002 of 2389 patients) had an estimated copy number greater than 2.0. The estimated BRF2 copy numbers ranged from 0.8 to 9.2 in 2389 patients diagnosed with IDC.

**Fig. 4:**
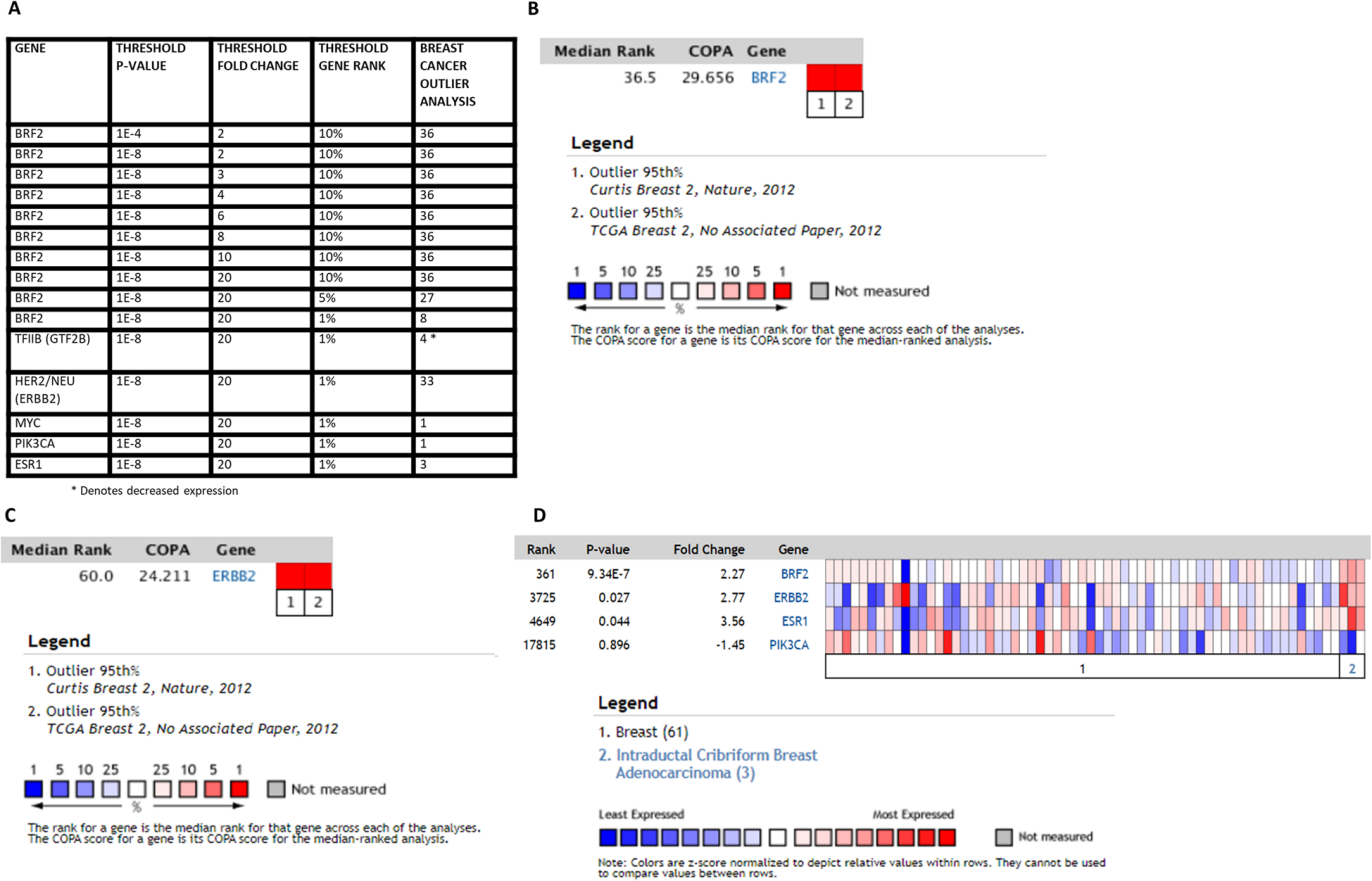
Cancer Outlier Profile Analysis (COPA) of BRF2 in breast cancer. Outlier analysis was performed to identify if BRF2 is an outlier in a subset of breast cancer samples using BRF2 gene expression data in Oncomine [21]. **a** Outlier analysis results for BRF2 in breast cancer using threshold p-value 1E-8, fold change of 20, and gene rank of top 1%. Outlier analysis of 1992 samples from the Curtis Breast 2 [27] and 1602 samples from the TCGA Breast 2 data sets [28, 34, 35] was performed for (**b**) BRF2 and (**c**) ERBB2 (HER2) to determine median gene rank and COPA score. (**d**) Comparison of fold-change in expression of BRF2 and a subset of known biomarkers in breast cancer using the TCGA Breast Cancer Data set [28], *n* = 593 samples. The data sets analyzed during the current study are available at www.oncomine.org. Queries of Oncomine were performed in September 2019, October 2019, November 2019, January 2020, February 2020, and March 2020

The Outlier analysis of human cancers in Fig. 1b identified 36 outlier breast cancer data sets with significant BRF2 overexpression. Outlier analysis and COPA are important to identify potential oncogenes because of the molecular heterogeneity in cancers. We repeated the Outlier analysis under increasingly stringent criterion and increased the threshold *p*-value to 1E-8, the threshold fold change in gene expression to 20, and the threshold gene rank to the top 1% of genes. Under these highly stringent selection criterion, the Outlier analysis returned 8 breast cancer data sets where BRF2 is significantly overexpressed (Fig. 4a). Next, we compared the BRF2 Outlier results to known breast cancer biomarkers, HER2 (ERBB2) [36], MYC [37], PIK3CA [38], and ER (ESR1) [39] using the same stringent threshold criterion (Fig. 4a). The outlier analysis identified HER2 as overexpressed in 33 breast cancer data sets and ESR1 as overexpressed in 3 breast cancer data sets whereas BRF2 is overexpressed in 8 breast cancer data sets. Performing the same Outlier analyses, MYC and PIK3CA are overexpressed in 1 breast cancer data set each. In contrast, TFIIB is underexpressed in 4 breast cancer data sets (Fig. 4a). In Oncomine, the Outlier analysis normalizes expression data making the median of each expression feature across all samples 0.0 and the mean absolute deviation is 1.0 [21]. Genes are then ranked based on their value at the 75th, 90th, or 95th percentiles in the Outlier analysis [21]. Using the results from the Outlier analyses for BRF2 (Fig. 1b and Fig. 4a) we analyzed BRF2 across outlier breast cancer data sets with greater than 1000 samples and restricted the analysis to the 95th percentile to determine median gene rank and COPA score for BRF2. For this COPA meta-analysis, the threshold p-value was set to 1E-8, the threshold fold change in gene expression to 20, and the threshold gene rank to the top 1% of genes. The meta-analysis included 1992 samples from the Curtis Breast 2 [27] and 1602 samples from the TCGA Breast 2 data sets [28] and identified a 36.5 median gene rank for BRF2 and a COPA score of 29.656 (Fig. 4b). Using the same data sets and criterion used for BRF2 in Fig. 4b, we analyzed HER2 (ERBB2) and report a median gene rank of 60 and a COPA score of 24.211 (Fig. 4c). Together, the median gene rank and COPA scores for BRF2 (Fig. 4b) and HER2 (Fig. 4c) are comparable in the meta-analysis of 3594 breast cancer patients included in this analysis. In Fig. 4d, we compare fold-change in expression for BRF2 and a subset of known biomarkers in breast cancer using the TCGA Breast Cancer Data set [28], *n* = 593 samples. The fold-change in expression for BRF2 (2.27-fold change, *p* = 9. 34E-7) and the breast cancer biomarker ERBB2 (HER2) (2.77-fold change, *p* = 0.027) are similar in the same data set.

**Fig. 5:**
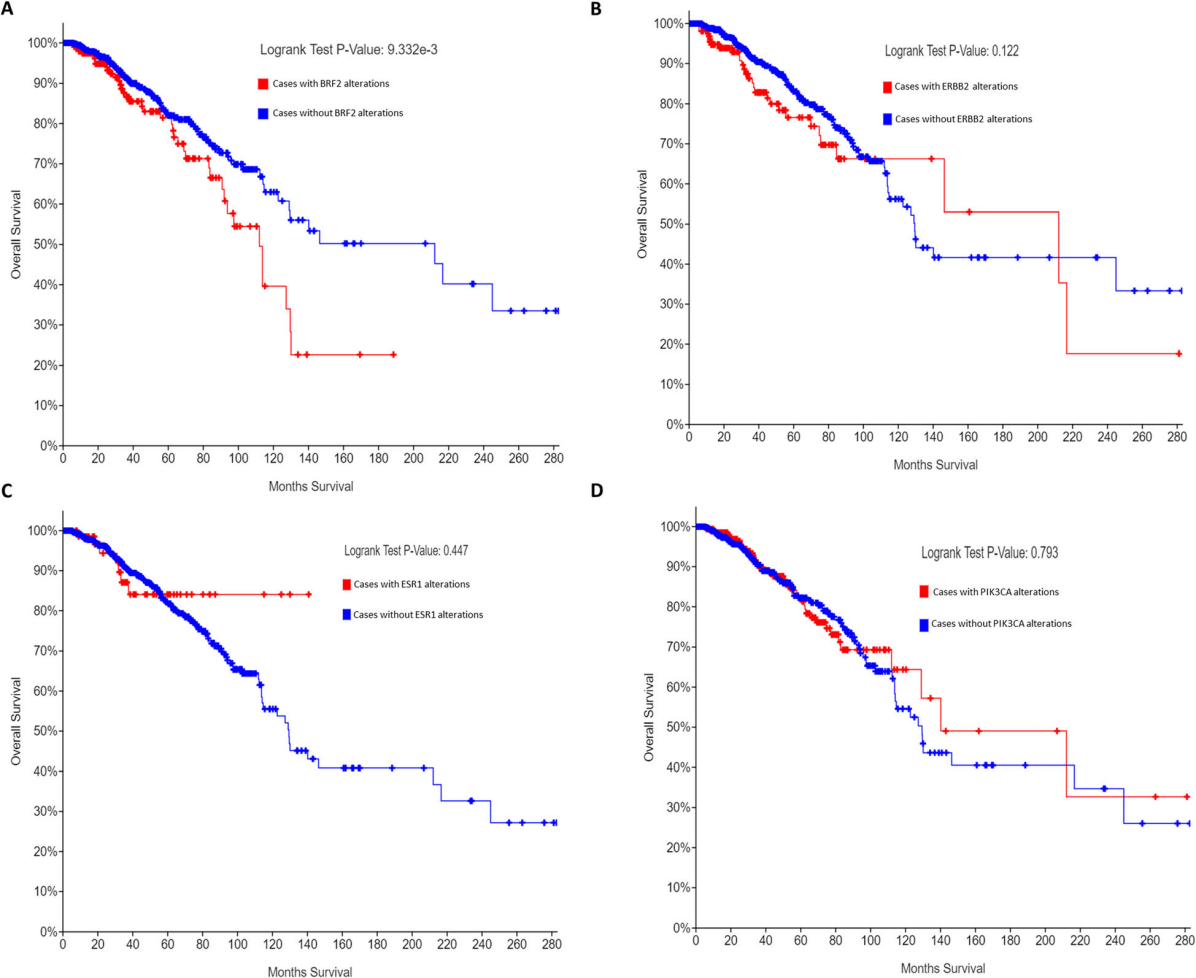
BRF2 is a prognostic marker for overall patient survival in invasive breast carcinoma. Using the cBioPortal [26], we queried a subset of the Invasive Breast Cancer Data Set (TCGA, Firehose Legacy) [28, 34, 35] for BRF2 alterations, restricting our analysis to complete patient data sets which included mutations, CNA, and mRNA microarray expression data (960 patients). BRF2 is altered in 21% of the patients queried and the overall survival Kaplan-Meier estimate was determined (**a**) comparing overall patient survival status, by month, in patients with BRF2 alterations (red line) to those without alterations in BRF2 (blue line). The logrank test p-value was 9.332e-3 and highly statistically significant. BRF2 overall survival status was compared to (**b**) ERBB2 (HER2), (**c**) ESR1 (ER), and (**d**) PIK3CA, known oncogenes and biomarkers. ERBB2 (HER2) (**b**) is altered in 18% of the patients queried with a logrank test p-value of 0.122. ESR1 (ER) (**c**) is altered in 9% of the patients queried with a logrank test p-value of 0.447. PIK3CA (**d**) is altered in 38% of patients queried with a logrank test p-value of 0.793. The Breast Invasive Carcinoma Data set (TCGA, Firehose Legacy) analyzed is available at the cBioPortal (www.cbioportal.org). Queries of the cBioPortal were performed in September 2019, October 2019, November 2019, January 2020, February 2020, and March 2020

BRF2 has been determined to be overexpressed in a subset of human cancers [8, 17, 19], but BRF2 alterations have never been investigated to correlate with clinical outcomes in breast cancer. In Oncomine, microarray expression data for breast cancer were analyzed [21], and overexpression of BRF2 was identified in the TCGA IBC data set [28], Fig. 1a. Thus, in our subsequent queries of the cBioPortal, we queried a subset of complete patients’ data in the TCGA IBC data set (TCGA, Firehose Legacy) [28]. The cBioPortal is a web-based platform for cancer bioinformatics queries; raw data normalization and subsequent algorithms used in these analyses are those designed by the cancer computational biologists and bioinformatics experts who designed and maintain the cBioPortal [26]. This strategy ensures experts in the RNA pol III/TFIIIB field may readily repeat the analyses performed in this study. The cBioPortal’s TCGA, Firehose Legacy IBC data set notes raw data was generated by the TCGA Research Network (https://www.cancer.gov/tcga), and source mutation data is pulled from the Broad Institute’s GDAC Firehose (https://gdac.broadinstitute.org/) [28]. In our current analysis of BRF2 in IBC, we included the 960 patients with mutation, CNA, and mRNA microarray expression (z-score threshold + 2.0) data available in cBioPortal’s TCGA, Firehose Legacy data set to identify if BRF2 alterations correlate with overall patient survival potentially. BRF2 is altered in 21% of the patients queried, 206 of 960 patients, with complete data as defined above. The majority of the BRF2 alterations are amplifications of copy number (7.7%) and an increase in BRF2 mRNA expression (3.85%), but multiple BRF2 alterations (5.31%) co-occur in IBC (data not shown). The Kaplan-Meier estimate of overall survival was determined by comparing overall patient survival status, by month, in IBC patients with BRF2 alterations (red line) to those without alterations in BRF2 (blue line) (Fig. 5a). The logrank test *p*-value is 9.332e-3 and is highly statistically significant. Next, we compared BRF2 overall survival status results with known oncogenes and biomarkers in breast cancer by analyzing the same data set for ERBB2 (HER2), ESR1 (ER), and PIK3CA. ERBB2 (HER2) (Fig. 5b) is altered in 18% of the breast invasive carcinoma patients queried, 177 of 960 patients, and the logrank test p-value is 0.122. ESR1 (ER) (Fig. 5c) is altered in 9% of the patients queried, 82 of 960, and the Kaplan-Meier estimate logrank test p-value is 0.447. PIK3CA (Fig. 5d) is altered in 368 of 960, 38%, IBC patients queried and the logrank test p-value is 0.793. Figure 5 suggests BRF2 alterations specifically and significantly correlate with overall patient survival in patients with IBC. No significant decrease in overall survival for patients with alterations in ERBB2 (HER2) (Fig. 5b), the ER (ESR1) (Fig. 5c), or PIK3CA (Fig. 5c) were observed. Patients with BRF2 alterations had a median survival of 111.99 months (*p* = 9.332e-3) as compared to a median survival of 212.09 months in patients without BRF2 alterations (Fig. 7a). In queries of the IBC (TCGA, Firehose Legacy) data set [28] in the cBioPortal [26], the Disease/Progression-free Kaplan median months disease-free were not available for patients with BRF2 alterations.

**Fig. 6:**
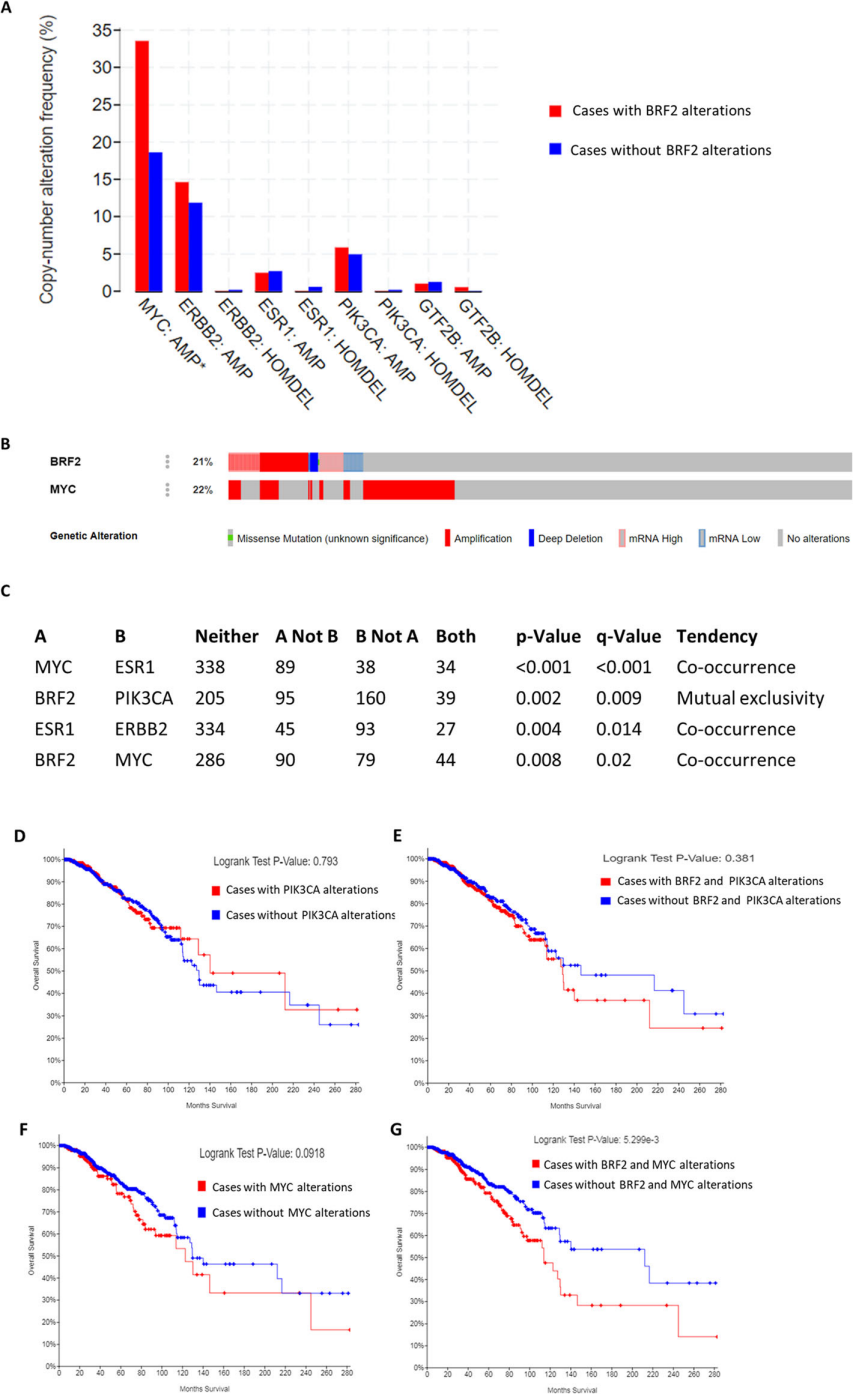
MYC and BRF2 alterations co-occur in breast invasive carcinoma and affect overall patient survival. **a** MYC is amplified in 35.5% of patients with BRF2 alterations, *p* = 6.638e-6. The breast cancer biomarkers ERBB2 (HER2), ESR1(ER), PIK3CA are not significantly altered in patients with BRF2 alterations. **b** OncoPrint of BRF2 and MYC alterations in the same data set analyzed in Fig. 7a. BRF2 and MYC are altered in 36% (346 of 960 patients, Breast Cancer Data Set, TCGA, Firehose Legacy) [28, 34, 35]. **c** BRF2 alterations tend to co-occur with MYC alterations (*p* = 0.008) and are mutually exclusive with PIK3CA alterations (*p* = 0.002). **d-g** Overall survival Kaplan-Meier estimate was determined comparing overall patient survival status, by month, in patients with alterations (red line) to those without alterations (blue line) in PIK3CA (**d**), BRF2 and PIK3CA (E), MYC (**f**), BRF2 and MYC (**g**). Logrank p-value for each overall survival Kaplan-Meier estimated is noted. The Breast Invasive Carcinoma Data set (TCGA, Firehose Legacy) analyzed is available at the cBioPortal (www.cbioportal.org). Queries of the cBioPortal were performed in September 2019, October 2019, November 2019, January 2020, February 2020, and March 2020

To elucidate if BRF2 alterations co-occur with alterations in known oncogenes and biomarkers in IBC we analyzed copy-number alteration frequency in breast cancer patients with BRF2 alterations (Fig. 6a). MYC has been demonstrated to regulate RNA pol III transcription [40] through the TFIIIB subunits BRF1 [11, 40, 41] and TBP [40, 42]. To the best of our knowledge MYC has never been experimentally determined to regulate BRF2 in breast cancer. MYC is frequently amplified in breast cancer and amplification is associated with poor prognosis [10, 37, 41]. MYC is amplified in 35.5% of patients with BRF2 alterations, *p* = 6.638e-6 (Fig. 6a). The breast cancer biomarkers ERBB2 (HER2), ESR1(ER), PIK3CA are not significantly altered in patients with BRF2 alterations (Fig. 6a). Figure 6b denotes OncoPrint of BRF2 (21%) and MYC (22%) alterations in the same data set analyzed in Fig. 5 and Fig. 6a. BRF2 and MYC are altered in 36%, 346 of 960 IBC patients (Fig. 6b).

Co-occurrence of gene alterations raise the possibility that together, they may contribute to specific cancer subtypes. To determine if the observed alterations in BRF2 and MYC are mutually exclusive or co-occur in breast invasive carcinoma we queried the IBC data set, TCGA, Firehose Legacy [28] in the cBioPortal [26]. BRF2 alterations tend to co-occur with MYC alterations (*p* = 0.008), Fig. 6c. In Fig. 6c, we determined if BRF2 alterations also co-occurred or are mutually exclusive with well-established breast cancer biomarkers. BRF2 alterations tend to be mutually exclusive from PIK3CA alterations (*p* = 0.002), Fig. 6c.

The data detailed in Fig. 6a-c, prompted an examination of overall survival for patients with breast invasive carcinoma with concurrent alterations in BRF2 and breast cancer biomarkers which were determined to be either co-occurring or mutually exclusive and statistically significant, Fig. 6c. Kaplan-Meier estimate was determined comparing overall patient survival status, by month, in patients with alterations (red line) to those without alterations (blue line) in PIK3CA (Fig. 6d), BRF2 and PIK3CA (Fig. 6e), MYC (Fig. 6f), BRF2 and MYC (Fig. 6g). Logrank *p*-value for each overall survival Kaplan-Meier estimated is noted. Patients with BRF2 and MYC alterations, Fig. 6g, had a median survival of 113.73 months (*p* = 5.299e-3) as compared to a median survival of 212.09 months in patients without BRF2 and MYC alterations. These data suggest co-occurring alterations in BRF2 and MYC significantly decrease overall survival in patients with breast invasive carcinoma. MYC and ESR1(ER) alterations tend to co-occur in breast invasive carcinoma (*p* < 0.001), Fig. 6c. The overall survival for alterations in MYC alone were significant as previously reported (Fig. 6f, *p* = 0.0918), ESR1 (ER) (*p* = 0.447), MYC and ESR1 (ER) (*p* = 0.263), ERBB2 (*p* = 0.122), ERBB2 and ESR1 (ER) (*p* = 0.305) were not statistically significant (data not shown).

**Fig. 7:**
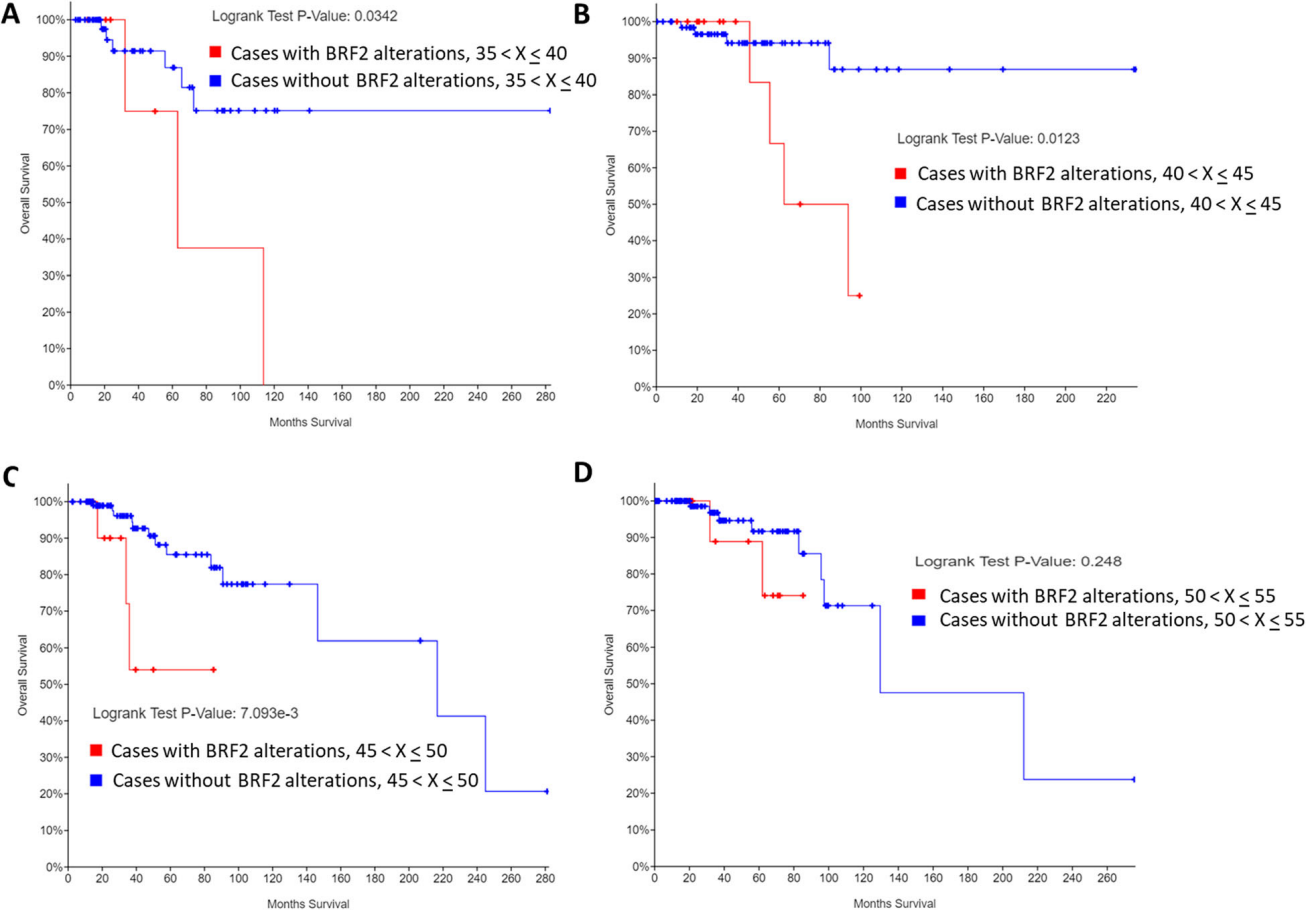
BRF2 alterations alter overall patient survival in an age-dependent manner. Using the cBioPortal [26], we queried a subset of the Invasive Breast Cancer Data Set (TCGA, Firehose Legacy) [28, 34, 35] for BRF2 alterations, restricting our analysis to complete patient data sets which included mutations, CNA, and mRNA microarray expression data (960 patients). Overall survival Kaplan-Meier estimate was determined comparing patients overall survival status, by month, in patients with alterations (red line) to those without alterations (blue line) in BRF2 analyzed by age of diagnosis: (**a**) 35 < X < 40, (**b**) 40 < X < 45, (**c**) 45 < X < 50, and (**d**) 50 < X < 55. Logrank p-value for each overall survival Kaplan-Meier estimated is noted. The Breast Invasive Carcinoma data set analyzed is available at the cBioPortal (www.cbioportal.org). Queries of the cBioPortal were performed in September 2019, October 2019, November 2019, January 2020, February 2020, and March 2020

Next, we investigated if age of first IBC diagnosis correlated with BRF2 alterations and overall survival outcome as BRF2 alterations significantly correlated with poor survival (*p* = 9.332e-3), Fig. 7. In this analysis only first-time IBC without evidence of metastasis were included from the TCGA, Firehose Legacy [28] data set in cBioPortal [26]. The overall Kaplan-Meier estimate was determined by comparing overall patient survival status, by month, in patients with BRF2 alterations (red line) to those without BRF2 alterations (blue line) by selected age ranges. 12% of women aged 35 < X < 40 (*n* = 59), had alterations in BRF2, classified as amplification in IBC, and have an overall decrease in patient survival (*p* = 0.032), Fig. 7a. In patients aged 40 < X < 45 (*n* = 81), BRF2 is altered (17%) and these alterations are overwhelmingly amplifications in IBC and correlated with overall patient survival (*p* = 0.0123), Fig. 7b. 13% of patients aged 45 < X < 50 (*n* = 121) have amplifications in BRF2 in both IBC and invasive lobular carcinoma (ILC), Fig. 7c. The decrease in overall survival of patients aged 45 < X < 50 (*n* = 121) was highly statistically significant (*p* = 7.093e-3). This was a noteworthy observation as most diagnoses of IBC occur in postmenopausal women [1, 2]. However, in our analysis of women with BRF2 alterations (13%) aged 50 < X < 55 (*n* = 119) showed no significant decrease in overall survival, Fig. 7d (*p* = 0.248). Survival in women with BRF2 alterations (17%) aged 55 < X < 60 (*n* = 118) were unaffected (*p* = 0.305), data not shown. Data from Figs. 5 and 7 suggest BRF2 may serve as a prognostic biomarker in IBC. Further, BRF2 alterations may be useful as an age-related prognostic marker for women, aged 35 < X < 50, with a first-time diagnosis of IBC or ILC. However, additional clinical studies are needed to ascertain the value of BRF2 as an age-related prognostic factor in IBC and ILC.

**Fig. 8:**
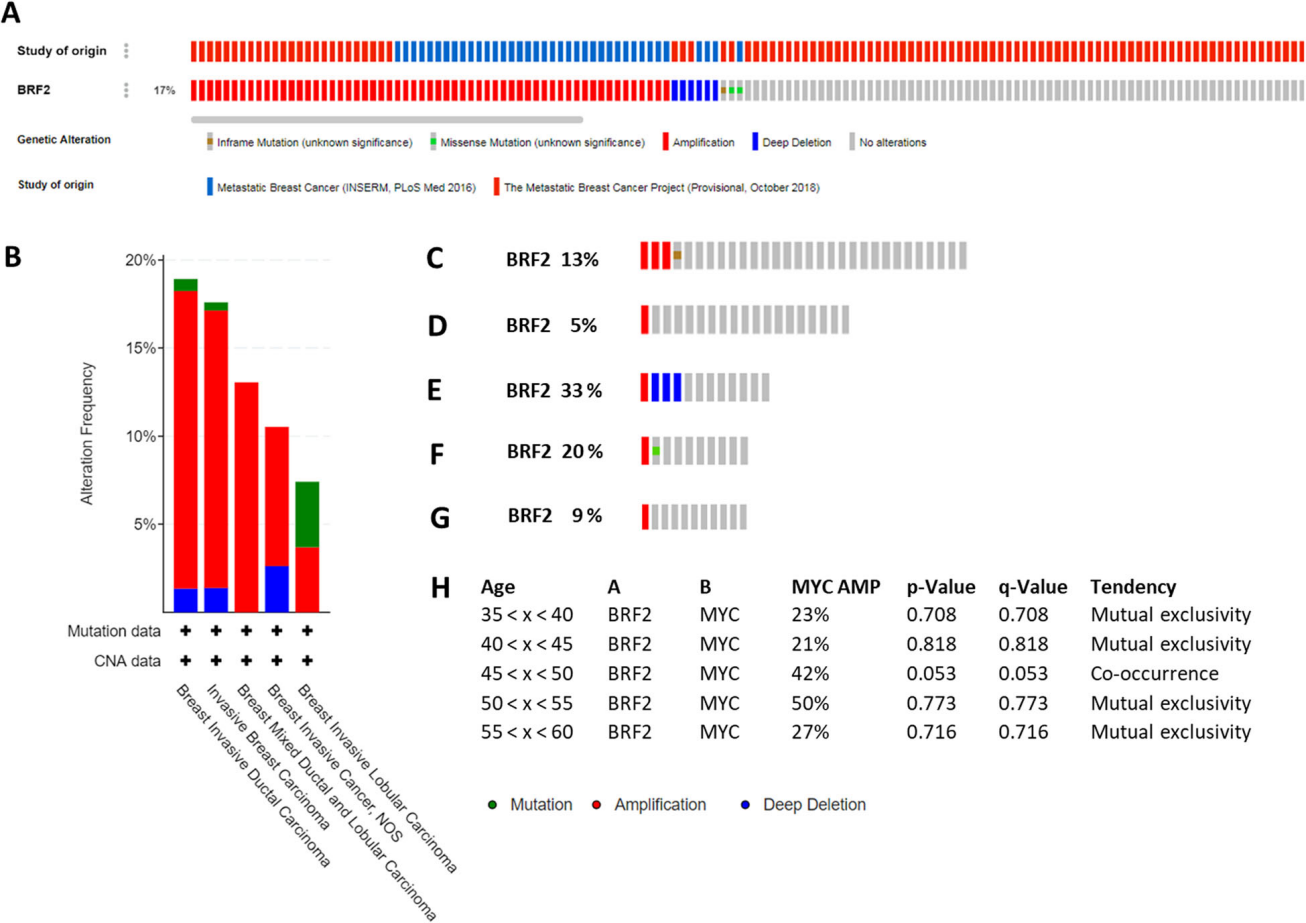
33% of women aged 45 < X < 50 have a deletion as the dominant BRF2 alteration in metastatic breast cancer. The cBioPortal houses two metastatic breast cancer data sets, The Metastatic Breast Cancer Project (Provisional, October 2018) [44] and Metastatic Breast Cancer (INSERM, PLoS Med 2016) [43]. A query of the metastatic breast cancer data sets, 396 patients, for BRF2 alterations was performed and (**a**) OncoPrint of BRF2 alterations indicates 17% (68 of 396 patients) of metastatic breast cancer patients analyzed have BRF2 alterations and the alterations are predominantly amplifications. Top track is color coded and denotes study of origin. (**b**) Summary of BRF2 alterations by breast cancer type. OncoPrint analysis of BRF2 alterations by age of primary breast cancer diagnosis grouped in 5-year intervals: (**c**) 35 < x < 40, (**d**) 40 < x < 45, (**e**) 45 < x < 50, (**f**) 50 < x < 55, (**g**) and 55 < x < 60. Red denotes amplification, blue represents deletions, and green indicates a mutation (H) Analysis of BRF2 and MYC alterations and tendency for co-occurrence, by age. *P*-Value and adjusted q-values are noted. Red denotes amplification, blue represents deletions, and green indicates a mutation. Metastatic breast cancer data sets analyzed are available at the cBioPortal (www.cbioportal.org). Queries of the cBioPortal were performed in September 2019, October 2019, November 2019, January 2020, February 2020, and March 2020

To the best of our knowledge, BRF2 alterations in metastatic breast cancer have not been investigated. Using the cBioPortal [26] we queried for BRF2 alteration in the two available metastatic breast cancer data sets: Metastatic Breast Cancer (INSERM, PLoS Med 2016) [43] and the Metastatic Breast Cancer Project (Provisional, October 2018) [44]. BRF2 is altered in 17%, 68 of 396, of patients with metastatic breast cancer, Fig. 8a. In these metastatic breast cancer data sets, BRF2 alterations are predominately amplifications, 14.57%. BRF2 alterations were identified in subtypes of metastatic breast invasive carcinoma, including IDL, IBC, breast mixed ductal and lobular carcinoma, and IBC NOS, Fig. 8b. Patient overall survival data is not available in these metastatic breast cancer data sets. However, we were able to query the metastatic data sets in the cBioPortal [26] for BRF2 alterations by age of primary breast cancer diagnosis grouped in 5-year intervals: (Fig. 8c) 35 < x < 40, (Fig. 8d) 40 < x < 45, (Fig. 8e) 46 < x < 50, (Fig. 8f) 50 < x < 55, (Fig. 8g) and 55 < x < 60. The highest frequency of BRF2 alterations, 33%, occur in women with metastatic breast cancer aged 45 < x < 50, Fig. 8e. These data prompted us to determine if BRF2 and MYC alterations tend to co-occur in women with metastatic breast cancer aged 46 < x < 50 (Fig. 8h), as observed in women aged 45 < x < 50 who did not have metastatic breast cancer (Fig. 6c). MYC and BRF2 alterations specifically co-occur in women aged 45 < x < 50 (*p* = 0.053) with metastatic breast cancer, Fig. 8h. MYC and BRF2 alterations in women not aged 45 < x < 50 analyzed in this meta-analysis tended to be mutually exclusive and were not statistically significant, Fig. 8h. Interestingly, deletions in BRF2 have been identified in the 33% of women aged 45 < x < 50, Fig. 8e. In this age range, deletions were the most common form of BRF2 alteration identified. It has not been previously identified or reported for BRF2 in metastatic breast cancer to the best of our knowledge. The data presented in this study agree with previous data demonstrating that 8p deletions have been linked to advanced tumor stage, MYC amplification, inversely associated with ER receptor expression, and shortened overall survival [45]. Further analysis of the patients with BRF2 deletions had clinical data available, which is correlative. For example, all these patients with BRF2 deletions are ages 45 < x < 50, self-reported race as white, were diagnosed as III high grade (poorly differentiated) and had negative ER and PR status per the medical record (MedR) diagnostic. HER2 status was not correlative. ER and PR negative breast cancers are classified as more aggressive [46]. Interestingly, 10–15% of ER negative breast cancers are responsive to tamoxifen treatment [47]. BRF2 has been demonstrated to be specifically regulated by the phytoestrogens epigallocatechin gallate (EGCG) and daidzein in cell culture and a mouse model [18, 19]. Together, these data suggest additional studies are warranted to determine if tamoxifen treatment would be beneficial in patients with IBC and BRF2 alterations.

## Discussion

BRF2 amplification and overexpression occur in a variety of human cancers, Fig. 1a, reviewed in [8]. Since the Cabarcas and Schramm Oncomine publication of 2011 [8], Oncomine 4.5 has more than doubled its data sets to 715 and 86, 733 samples [21]. In this study, we demonstrate BRF2 overexpression in cancers of the breast, stomach, kidney, skin, and sarcoma (Fig. 1a). Outlier analyses of the Oncomine microarray data suggest BRF2 is a novel outlier gene in breast cancer (Fig. 1). Notably, there is a 2.3-fold increase in BRF2 mRNA expression in Intraductal Cribriform Breast Adenocarcinoma, a form of invasive breast carcinoma, as compared to controls, *p* = 9.34E-7 (Fig. 1d). Together, these data prompted a more detailed analysis of BRF2 alterations in IBC.

IBC accounts for 80% of breast cancer diagnoses; 276,480 new cases of IBC are anticipated in 2020 [1, 2]. Invasive breast cancer has at least 21 distinct histological subtypes and four different molecular subtypes [28, 48]. Of the Oncomine breast cancer data sets available for analysis, 2.0% have an increased frequency of BRF2 expression (Fig. 2a) and ductal carcinoma had the highest BRF2 overexpression frequency (Fig. 4b). Individual patient data from scatterplot detailed in Fig. 2c identified 9% of patients with IDC having a 10-fold increase in BRF2 expression. Often, gene amplification correlates with increased gene expression. The cytogenetic location of BRF2 is 8p11.23 and amplification of the short arm of chromosome 8 is a frequent feature of breast cancer cell lines and tumors [33]. In this study, we demonstrate that BRF2 is amplified in 5.9% of all breast cancers and 6.9% of all ductal breast carcinomas queried (Fig. 3a-b). The average estimated copy number for BRF2 in ductal carcinoma is 2.28, with 42% having an estimated copy number greater than 2.0. (Fig. 3c). It has been reported that HER2 is a predictor of overall survival in patients where HER2 is amplified from 2- to greater than 20-fold [49]. In this analysis, the estimated BRF2 copy numbers ranged from 0.8 to 9.2 in 2389 patients diagnosed with IDC, Fig. 3c. Taken together, these data highlight the need to investigate BRF2 copy number and overall patient survival in breast cancer in greater detail.

For the COPA meta-analysis performed in Fig. 4, the threshold *p*-value was set to 1E-8, the threshold fold change in gene expression to 20, and the threshold gene rank to the top 1% of genes. The meta-analysis included 1992 samples from the Curtis Breast 2 [27] and 1602 samples from the TCGA Breast 2 data sets [34] and identified a 36.5 median gene rank for BRF2 and a COPA score of 29.656 (Fig. 4b). We analyzed HER2 and report a median gene rank of 60 and a COPA score of 24.211 using the same data sets and stringency (Fig. 4c). The median gene rank and COPA scores for BRF2 (Fig. 4b) and HER2 (Fig. 4c) are comparable in the meta-analysis of 3594 breast cancer patients included in this analysis. The COPA analysis suggest BRF2 is behaving as an oncogenic driver in IBC. As such, we then queried for BRF2 alterations and clinical outcomes, specifically overall patient survival. A query of the TCGA Firehose Legacy data set [35] in the cBioPortal for BRF2 alterations and patient overall survival status demonstrated a statistically significant correlation, Fig. 5a. Notably, in patients with BRF2 alterations, 21%, overall survival was drastically decreased, *p* = 9.332e-3, Fig. 5a. In contrast, alterations in the known breast cancer biomarkers HER2 (ERBB2) (Fig. 5b), ER (ESR1) (Fig. 5c), and PIK3CA (Fig. 5d) showed no correlation with overall survival in the same invasive breast cancer data set we examined for BRF2 alterations. Overall survival was dramatically decreased in breast cancer patients with co-occurring alterations in BRF2 and MYC, *p* = 5.299e-3, Fig. 6g. Further refinement of the invasive breast carcinoma data set queried in this study, demonstrated the most significant decline in overall survival was for patients 45 < x < 50, *p* = 7.093e-3, Fig. 7c. These data suggest BRF2 may prove a useful prognostic marker in IBC.

Additional IBC studies with survival and progression/disease-free data must be undertaken. Many available clinical breast cancer studies available are not comparable in a meta-analysis due to differences in clinical data and study definitions associated with the data sets. For example, overall survival and survival data in studies have been defined differently by medical investigators. However, a separate query of the Breast Cancer METABRIC [29] data set in the cBioPortal for BRF2 alterations and overall survival was correlative. In patients aged 55–60, BRF2 was altered in 16% (36 of 229 patients) and median months survival in patients with alterations in BRF2 was 143.13 and 213.20, in patients with no alterations, *p* = 6.47e-3 (Supplemental Table 1). Together, these data suggest a larger investigation of the applicability of BRF2 as a prognostic marker in patients with IBC aged 46–60. Further query of the Breast Cancer METABRIC [29], for patients aged 55–60 with BRF2 alterations, ER positive status (*n* = 170) correlated with a decrease in overall survival, *p* = 7.87e-3, whereas ER negative status (*n* = 59) did not, *p* = 0.516 (Supplemental Table 1). ER status was determined by immunohistochemistry (IHC) in the Breast Cancer METABRIC [29] data set. All patients with BRF2 alterations were coded as postmenopausal in the METABRIC) [29] data set which differed from patients with BRF2 alterations in the TCGA, Firehose Legacy data set.

Our study presents data suggesting BRF2 alterations in patients aged 35 to 50 correlates with overall survival (Fig. 7). Interestingly, ER- status correlated with a decrease in overall patient status in patients 46–50, *p* = 9.028e-3, whereas ER+ status did not, *p* = 0.183 (data not shown). The decrease in overall survival in patients, 46–50, classified as ER- and with BRF2 alterations, correlated with postmenopausal status, *p* = 1.377e-4 (data not shown). It has been reported 20% of ER and PR status determination may be inaccurate as determined by immunohistochemistry [50]. Recently, retesting of ER and PR status on post-surgical specimens have been determined to be clinically relevant [50, 51]. A larger study of BRF2 alterations and ER status in breast cancer is needed to determine correlative value.

It has been reported that 5–10% of all ER- breast cancers respond to tamoxifen treatment [47]. Estradiol was demonstrated to inhibit the growth of triple-negative breast cancer (negative ER alpha, PR and HER2) when ER beta was present [52]. A query of the Comparative Toxicogenomics Database (http://ctdbase.org/, accessed February 2020) [53] for estradiol and BRF2, indicates BRF2 mRNA is increased in estradiol treated hepatoma cell lines [54]. The phytoestrogen daidzein induces BRF2 mRNA in breast cancer cell lines and a mouse model [19]. Also, the phytoestrogen EGCG downregulated BRF2 expression [18] and EGCG has been speculated to be an ER antagonist [55]. Together, studies regarding BRF2 expression and chemotherapeutic agents working through the ER are necessary in the context of breast cancer.

MYC is amplified in breast cancer [56] and has been reported to be overexpressed in 30–50% of high grade breast cancers [37]. Recently, it has been hypothesized that an oncogenic event, such as the mutation of c-MYC, is required to facilitate an aberrant increase in RNA pol III activity for the initiation and progression in a model of breast cancer [57]. There is evidence that MYC amplifies transcription of RNA pol III genes when RNA pol III genes are hypomethylated in breast cancer, specifically the nc886 gene, a short noncoding RNA, which has been shown to be occupied by BRF1 and deregulated in cancer [57]. MYC activates RNA pol III transcription [40] through its physical interactions with the TFIIB family member BRF1 and TBP [41]. The interaction between MYC and BRF1 occur via the amino-terminal 262 residue of MYC [40] and we have previously demonstrated that the BRF1 promoter contains a MYC/MAX binding site [17]. In this study, we demonstrate MYC and BRF2 alterations co-occur in breast invasive carcinoma, *p* = 0.008 (Fig. 8a-c), as well as patients aged 46–50 with metastatic breast cancer, *p* = 0.053 (Fig. 8H). In the cBioPortal Breast Invasive Carcinoma (TGCA, Firehose Legacy) [28] data set, MYC alterations correlated with a decrease in overall survival, *p* = 0.0918 (Fig. 8f) and co-occurring MYC and BRF2 alterations significantly decreased overall survival, *p* = 5.299e-3 (Fig. 8g). The cytogenetic locations of BRF2 (8p11.23) [33] and MYC (8q24.21) [58] are on a portion of chromosome 8 known to be amplified in breast cancer. Targeting MYC overexpression is a challenge in cancer treatment as MYC plays a pivotal role in cellular functions, including cell cycle progression, metabolism, cell adhesion, signal transduction, transcription, and translation, protein biogenesis. MYC overexpression may occur as a result of an increase in MYC mRNA stability [58]. MYC contains a coding region instability determinant (CRD), located in the last 249 nucleotides of the coding region, which promotes rapid mRNA turnover [59] and recruits a 68-kDa CRD-binding protein (CRD-BP) protecting MYC mRNA from turnover [60]. Recently, 2-[[(5-bromo-2-thienyl)methylene]amino]-benzamide (BTYNB) was identified as a selective inhibitor of CRD-BP binding to the CRD region of MYC mRNA thereby increasing MYC turnover in melanoma and ovarian cancer cells [61]. Previously, we determined that the CRD sequence of MYC (ntds 1707–1890) and the c-term coding region of BRF2 (ntds 1369–1257) are 50.1% identical [17]. Together, these data lead us to speculate a novel therapeutic target to decrease BRF2 overexpression in IBC may involve targeting the CRD region of BRF2.

Also, a query of the Eukaryotic Promoter Database for putative MYC binding sites in the BRF2 promoter [18] identified MYC sites at base pairs − 699, − 864, − 994 and − 995, relative to the transcriptional start site (TSS), *p*-value of 0.001, (https://epd.epfl.ch//index.php, accessed March 2020). This suggests MYC directly interacts with the BRF2 promoter and contributes to its overexpression.

## Conclusions

To the best of our knowledge, this is the first reported study to identify BRF2 as a prognostic factor for overall survival in patients with IBC using publicly available data sets. Breast cancer subtypes differ in terms of risk factors, presentation, response to treatment, and outcomes [62]. Based on the clinical data analyzed, we believe additional clinical investigations of BRF2 as a prognostic marker in IBC is warranted, especially for patients 35–50 (Fig. 7). The data presented suggest BRF2 may be a novel target for therapeutic intervention for patients with IBC.

## Supplementary Information

**Additional file 1: Table S1.** BRF2 alterations and ER status correlate with overall patient survival in patients aged 55 < x < 60 in the Breast Cancer METABRIC [29] data set.

## Data Availability

Data sets used in these analyses are available at www.oncomine.org and www.cbioportal.com.

## Abbreviations

3FLND: Three-field lymph-node dissection
BRCA1: Breast cancer type 1
BRF: TFIIB related factor
BTYNB: 2-[[(5-bromo-2-thienyl)methylene]amino]-benzamide
COPA: Cancer outlier profile analysis
CRD: Coding region instability determinant
CRD-BP: Coding region instability determinant binding protein
EGCG: Epigallocatechin gallate
ER: Estrogen receptor
ERBB2: Erb-b2 receptor tyrosine kinase 2 gene
ERK: Extracellular signal-regulated kinases
ESCC: Esophageal squamous cell carcinoma
ESR1: Estrogen receptor 1 gene
HER2: Human epidermal growth factor receptor 2
IBC: Invasive breast cancer
IDC: Invasive ductal carcinoma
ILC: Invasive lobular carcinoma
MAPK: Mitogen-activated protein kinase
MedR: Medical Record
MYC: Myelocytomatosis oncogene
NOS: Not otherwise specified (histological diagnosis)
NSCLC: Nonsmall cell lung cancer
PIK3CA: Phosphatidylinositol-4,5-bisphosphate 3-kinase catalytic subunit alpha
Pol: Polymerase
PR: Progesterone receptor
PTEN: Phosphatase and tensin homolog
RB: Retinoblastoma protein
SQCC: Squamous cell carcinoma
U.S.: United States
