# Supplementary material for: A meta-analysis of BRF2 as a prognostic biomarker in invasive breast carcinoma
Source: BMC Cancer. 2020 Nov 11;20:1093. doi: 10.1186/s12885-020-07569-8 (PMC7659115; doi:10.1186/s12885-020-07569-8)
Supplement: Supplementary file 1 — Additional file 1: Table S1. BRF2 alterations and ER status correlate with overall patient survival in patients aged 55 < x < 60 in the Breast Cancer METABRIC [29] data set. [file 12885_2020_7569_MOESM1_ESM.docx]

**Supplemental Data**

Table 1. BRF2 alterations and ER status correlate with overall patient survival in patients aged 55< x < 60 in the Breast Cancer METABRIC (29) data set.

| Sample size (ER Status) | Age range | % BRF2 alterations | ER status (IHC) | Logrank p-value for Kaplan-Meier Estimate |
| --- | --- | --- | --- | --- |
| 74 | 40 < x < 45 | 14% | positive | 0.0982 |
| 55 | 40 < x < 45 | 9% | negative | 0.955 |
| 112 | 45 < x < 50 | 13% | positive | 0.901 |
| 51 | 45 < x < 50 | 10% | negative | 0.688 |
| 142 | 50< x < 55 | 14% | positive | 0.177 |
| 58 | 50< x < 55 | 0% | negative | NA |
| 170 | 55< x < 60 | 16% | positive | 7.87e-3 |
| 53 | 55< x < 60 | 19% | negative | 0.272 |
| 223 | 60< x < 65 | 22% | positive | 0.775 |
| 48 | 60< x < 65 | 8% | negative | 0.543 |
